# Timing of Biliary Drainage and Clinical Outcomes in Acute Cholangitis: A Single-Center Retrospective Cohort Study

**DOI:** 10.7759/cureus.105350

**Published:** 2026-03-16

**Authors:** Ayano Nakamura, Kou Watanabe, Wataru Sato, Keito Kishimoto, Kensaku Hiyajo, Yuta Kawakami, Yutaro Uchida, Kota Fukumine, Yoshiki Nakamura, Munehiro Mayama, Ryo Kimura, Yumi Takamori, Takashi Shinzato, Yasuharu Tokuda

**Affiliations:** 1 Division of Medical Residency Program, Nakagami Hospital, Okinawa, JPN; 2 Department of General Internal Medicine, Nakagami Hospital, Okinawa, JPN; 3 Internal Medicine, Muribushi Project for Okinawa Residency Programs, Okinawa, JPN

**Keywords:** acute cholangitis, biliary drainage, endoscopic retrograde cholangiopancreatography, functional outcomes, patient-centered outcomes, severity grading, tokyo guidelines

## Abstract

Background and aims: Although guidelines recommend early biliary drainage for acute cholangitis, evidence for optimal timing across severity grades remains limited. We examined associations between drainage timing and both functional and survival outcomes stratified by disease severity using the Tokyo Guidelines 2018 criteria.

Methods: This retrospective observational cohort study included 214 consecutive patients with acute cholangitis who underwent endoscopic retrograde cholangiopancreatography between April 2022 and March 2024 at a tertiary center in Okinawa, Japan. Patients were classified according to the 2018 Tokyo Guidelines severity criteria. The primary outcome was discharge disposition (favorable: discharge to pre-admission residence; unfavorable: alternative facility or death). Time from presentation to drainage was analyzed at 12-, 24-, 36-, and 48-hour cutoffs using Fisher's exact test.

Results: The cohort comprised 92 Grade I (43%), 83 Grade II (39%), and 39 Grade III (18%) patients. Discharge-to-pre-admission-residence rates were 95% (87/92), 80% (66/83), and 69% (27/39), respectively. For Grade I, drainage within 24 hours was associated with 100% (59/59) discharge to pre-admission residence versus 88% (28/32) beyond 24 hours (P = 0.013). Grade II showed no functional benefit but was associated with improved survival when drainage occurred within 48 hours (99% vs 82%, P = 0.045). Grade III survivors showed better functional recovery with drainage within 24 hours (92% vs 50%, P = 0.014), though this comparison included only 10 patients in the delayed group and should be interpreted with considerable caution.

Conclusions: Associations between drainage timing and outcomes varied by severity grade and outcome type. Given the observational design and limited adjustment for confounders, these findings suggest severity-stratified drainage protocols may be beneficial but require validation through prospective studies with multivariable adjustment to establish causality.

## Introduction

Acute cholangitis is a potentially life-threatening biliary infection requiring prompt antimicrobial therapy and biliary drainage [1]. The overall mortality from acute cholangitis is less than 10% after early biliary drainage, and severity and timely intervention remain crucial for optimal outcomes [1]. Despite advances in management, mortality from acute cholangitis remains approximately 5% [2].

The Tokyo Guidelines 2018 recommend urgent drainage for severe cholangitis and early drainage for moderate cases [3]. However, heterogeneity in drainage timing across institutions was observed, with median times ranging from 12 to 72 hours [4-8]. Therefore, evidence for specific hour-based targets remains limited. In addition, clinical practice in the real world varies considerably due to institutional factors, including staffing patterns, resource availability, and the challenges of off-hour presentations [9]. 

Most previous studies have focused primarily on mortality outcomes. However, functional recovery, particularly discharge to the pre-admission residence, is increasingly recognized as a patient-centered outcome in older populations [10,11]. This outcome reflects not only survival but also preservation of functional independence and quality of life. Whether optimal drainage timing for acute cholangitis differs across severity grades when considering functional outcomes alongside survival remains unclear.

We conducted a retrospective observational cohort study to examine associations between severity-specific timeframes for biliary drainage (at 12-, 24-, 36-, and 48-hour cutoffs) and both functional recovery (discharge to pre-admission residence) and in-hospital survival in patients with acute cholangitis stratified by Tokyo Guidelines 2018 severity grades.

## Materials and methods

Study design and population

We retrospectively reviewed all patients diagnosed with acute cholangitis (ICD-10 K83.0) undergoing endoscopic retrograde cholangiopancreatography at our tertiary center between April 1, 2022, and March 31, 2024. The study was approved by the Ethics Review Board of Muribushi Okinawa Center for Teaching Hospitals (IRB #2024-5) with waiver of informed consent due to its retrospective nature.

Inclusion criteria were: (1) diagnosis of acute cholangitis according to Tokyo Guidelines 2018, (2) age 18 years or older, and (3) endoscopic biliary drainage performed. Exclusion criteria were: (1) previous drainage procedure within 30 days, (2) stent exchange or removal only without new drainage, and (3) percutaneous or surgical drainage as primary intervention. Patients with multiple admissions during the study period had only their first admission included.

Data collection

Five reviewers independently extracted data from electronic medical records, with discrepancies resolved by consensus. The variables extracted included: age, sex, comorbidities, vital signs at presentation, laboratory values (complete blood count, hepatic and renal function tests, coagulation profile, blood cultures), imaging findings, procedural details, time from presentation to drainage, complications, length of stay, antibiotic duration, and discharge disposition.

Clinical variables at presentation were defined as follows. Hepatic dysfunction was defined as total bilirubin ≥ 5 mg/dL or elevation of hepatic transaminases (aspartate aminotransferase (AST) or alanine aminotransferase (ALT)) above 1.5 times the upper limit of normal. Sepsis was defined according to the Sepsis-3 criteria (suspected infection with Sequential Organ Failure Assessment score ≥ 2) [12]. Acute kidney injury was defined according to the Kidney Disease: Improving Global Outcomes (KDIGO) criteria (increase in serum creatinine ≥ 0.3 mg/dL within 48 hours or ≥ 1.5 times baseline) [13]. Disseminated intravascular coagulation was identified using the International Society on Thrombosis and Haemostasis scoring system [14].

Data completeness was assessed for all variables; missing data were minimal (< 2% for any single variable) and were handled by complete case analysis.

Severity classification

Patients were classified according to the Tokyo Guidelines 2018 [15,16]. Grade I (Mild) was defined as acute cholangitis responding to initial medical treatment without organ dysfunction. Grade II (Moderate) was defined as acute cholangitis with two or more of the following: abnormal white blood cell count (greater than 12,000 or less than 4,000/µL), fever 39°C or higher, age 75 years or older, total bilirubin 5 mg/dL or greater, or albumin less than 3.0 g/dL. Grade III (Severe) was defined as acute cholangitis with cardiovascular, neurological, respiratory, renal, hepatic, or hematological organ dysfunction.

Outcomes

The primary outcome was discharge disposition, dichotomized into “favorable” (discharge to pre-admission residence, including home, assisted living, or nursing facility from which admitted) and “unfavorable” (discharge to an alternative facility or in-hospital mortality).

Statistical analysis

Time to drainage was calculated from emergency department presentation time to the procedure start time, then categorized at clinically relevant cutoffs of 12, 24, 36, and 48 hours. Categorical variables were compared using Fisher's exact test, continuous variables using the Mann-Whitney U test for two-group comparisons or Dunn's post hoc test with Bonferroni correction.

Multivariable logistic regression was not performed due to limited outcome events (Grade I: n=5 unfavorable; Grade II: n=17; Grade III: n=12), which would yield unstable estimates with fewer than 10 events per variable [17]. 

Statistical significance was set at *P* < 0.05. All *P*-values are two-tailed. Analysis was performed using Python 3.13.1, statsmodels 0.14.0. 

## Results

Patient characteristics

Of 248 patients undergoing drainage procedures for acute cholangitis during the study period, 214 met the inclusion criteria (Table 1). Median age was 82 years, with 49% (105/214) male. Primary etiologies included choledocholithiasis in 63% (135/214), malignant biliary obstruction in 16% (34/214), and benign strictures in 2% (5/214).

**Table 1:** Clinical characteristics of 214 patients with acute cholangitis ^†^ Including IgG4-related sclerosing cholangitis, peripapillary diverticulum, pericholecystic abscess, stent occlusion, and stent dislocation. ^‡^ Counting overlapping cases.

Characteristics	n (%)
Age, median (range)	82 (36 – 98)
Male gender	105 (49)
Underlying diseases	
Cholecystolithiasis	135 (63)
Tumors obstruction	34 (16)
Others^†^	5 (2)
None	40 (19)
Complications^‡^	
Hepatic dysfunction	191 (89)
Sepsis/Septic shock	105 (49)
Acute kidney injury	52 (24)
Disseminated intravascular coagulation	5 ( 2)
None	23 (11)
Result of pre-treatment blood culture	
Positive	105 (49)
Negative	104 (49)
Not performed	5 ( 2)

At presentation, 89% (191/214) had evidence of hepatic dysfunction, 49% (105/214) met sepsis criteria, 24% (52/214) had acute kidney injury, and 2% (5/214) had disseminated intravascular coagulation. Blood cultures were positive in 49% (105/214) of patients.

Severity distribution and outcomes

The cohort comprised 92 Grade I (43%), 83 Grade II (39%), and 39 Grade III (18%) patients (Table 2). Drainage within 24 hours was achieved in 74% (29/39) of Grade III, 66% (55/83) of Grade II, and 64% (59/92) of Grade I patients.

**Table 2:** Timing of endoscopic biliary drainage and clinical outcome according to cholangitis severity grade ^†^ Grade I vs. Grade II: *P *= 0.006, Grade I vs Grade III: *P* = 0.002, Grade II vs Grade III: *P* = 1.00. ^‡^ Grade I vs Grade II: *P* = 0.009, Grade I vs Grade III: *P* = 0.003, Grade II vs Grade III: *P* = 0.36. Dunn's test with a Bonferroni correction was performed for multiple comparisons of the group means. Statistically significant difference (*P *< 0.05).

	Grade I n=92 (%)	Grade II n=83 (%)	Grade III n=39 (%)
Timing to endoscopic biliary drainage			
≤12 h	35 (38)	30 (36)	22 (56)
12 - 24 h	24 (26)	25 (30)	7 (18)
24 - 36 h	14 (15)	13 (16)	3 ( 8)
36 - 48 h	10 (11)	4 ( 5)	2 ( 5)
> 48 h	9 (10)	11 (13)	5 (13)
Outcome			
Days of hospital stay^†^	10.0±7.7	15.8±16.4	19.2±20.0
Duration of antibiotic therapy^‡^	8.7±6.3	11.1±7.0	13.1±12.4
Recovered and returned	87 (95)	66 (80)	27 (69)
Recovered but not returned	4 ( 4)	14 (17)	7 (18)
In-hospital mortality	1 ( 1)	3 ( 4)	5 (13)

Discharge to pre-admission residence rates declined significantly with increasing severity: 95% (87/92) in Grade I, 80% (66/83) in Grade II, and 69% (27/39) in Grade III. In-hospital mortality increased from 1% (1/92) in Grade I to 4% (3/83) in Grade II and 13% (5/39) in Grade III. Grade III patients had significantly more extended hospital stays (mean 19.2 ± 20.0 vs 10.0 ± 7.7 days for Grade I, *P* = 0.0023) and longer antibiotic courses (13.1 ± 12.4 vs 8.7 ± 6.3 days, *P* = 0.0028).

Drainage timing and outcomes by severity

Table 3 presents the relationship between drainage timing and outcomes by severity grade. Among Grade I survivors (n=91), drainage within 24 hours was associated with 100% (59/59) discharge to pre-admission residence versus 88% (28/32) for drainage beyond 24 hours (*P* = 0.013). Similar associations were observed at 36-hour (99% vs 83%, *P* = 0.023) and 48-hour (98% vs 75%, *P* = 0.037) cutoffs. No mortality difference was observed.

**Table 3:** Impact of drainage timing on functional recovery and survival by severity grade ^†^Fisher's exact test was performed for comparisons of the groups between early (≤ threshold) and late (> threshold) drainage timing. ^‡^ Statistically significant difference (*p* < 0.05).

Timing to endoscopic biliary drainage	Functional recovery in survivors n (%)			Survival n (%)		
	Early	Late	*p* value^†^	Early	Late	*p* value^†^
Grade I						
≤ 12h vs > 12h	35/35 (100)	52/56 (93)	0.290	35/35 (100)	56/57 (98)	1.0
≤ 24h vs > 24h	59/59 (100)	28/32 (88)	0.013^‡^	59/59 (100)	32/33 (97)	0.360
≤ 36h vs > 36h	72/73 (99)	15/18 (83)	0.023^‡^	73/73 (100)	18/19 (95)	0.210
≤ 48h vs > 48h	81/83 (98)	6/8 (75)	0.037^‡^	83/83 (100)	8/9 (89)	0.098
Grade II						
≤ 12h vs > 12h	24/29 (83)	42/51 (82)	1.0	29/30 (97)	51/53 (96)	1.0
≤ 24h vs > 24h	47/54 (87)	19/26 (73)	0.210	54/55 (98)	26/28 (93)	0.260
≤ 36h vs > 36h	56/67 (84)	10/13 (77)	0.690	67/68 (99)	13/15 (87)	0.083
≤ 48h vs > 48h	59/71 (83)	7/9 (78)	0.650	71/72 (99)	9/11 (82)	0.045^‡^
Grade III						
≤ 12h vs > 12h	17/19 (89)	10/15 (67)	0.200	19/22 (86)	15/17 (88)	1.0
≤ 24h vs > 24h	22/24 (92)	5/10 (50)	0.014^‡^	25/29 (86)	9/10 (90)	1.0
≤ 36h vs > 36h	24/28 (86)	3/6 (50)	0.086	28/32 (88)	6/7 (86)	1.0
≤ 48h vs > 48h	25/30 (83)	2/4 (50)	0.100	30/34 (88)	4/5 (80)	0.520
Overall						
≤ 12h vs > 12h	76/83 (92)	104/122 (85)	0.180	83/87 (95)	122/127 (96)	1.0
≤ 24h vs > 24h	128/137 (93)	52/68 (76)	0.006^‡^	137/143 (96)	68/71 (96)	1.0
≤ 36h vs > 36h	152/168 (90)	28/37 (76)	0.038^‡^	168/173 (97)	37/41 (90)	0.210
≤ 48h vs > 48h	165/184 (90)	15/21 (71)	0.048^‡^	184/189 (97)	21/25 (84)	0.043^‡^

Grade II patients showed no significant functional recovery benefit from earlier drainage at any time point. However, drainage within 48 hours was associated with improved survival (99% vs 82%, *P* = 0.045). The functional recovery rate among survivors was similar regardless of timing (83% early vs 78% late, *P* = 0.650).

Among Grade III survivors (n=34), drainage within 24 hours was associated with better functional recovery (92% [22/24] vs 50% [5/10], *P* = 0.014). However, this finding is based on only 10 patients in the delayed group, resulting in limited statistical power, and should be interpreted with considerable caution as a hypothesis-generating study. No significant difference in survival was observed (86% vs 90%, *P* = 1.00).

Overall, across all grades, drainage within 24, 36, and 48 hours was associated with higher functional recovery among survivors (93% vs. 76%, *P* = 0.006; 90% vs. 76%, *P* = 0.038; 90% vs. 71%, *P* = 0.048, respectively). The 12-hour cutoff was not significant (92% vs. 85%, *P* = 0.180).

## Discussion

In this single-center retrospective observational analysis of 214 patients with acute cholangitis, we found that the association between biliary drainage timing and clinical outcomes varied by disease severity and outcome type. The findings in this study partially align with recent meta-analyses, which show mortality benefits from drainage within 24-48 hours [4,18,19]. In addition, the primary outcome represents a novel, patient-centered approach that suggests it could be beyond mere survival and provide meaningful information for patients and families.

Our findings should be interpreted in the context of other single-center retrospective analyses examining drainage timing. Zhu et al. used the MIMIC-III database to study 177 ICU patients with severe acute cholangitis and found that delayed biliary drainage increased hospital and ICU length of stay, though drainage within 24 hours did not significantly reduce mortality [20]. Similarly, Aboelsoud et al. analyzed 177 critically ill patients from the same database and reported that early drainage within 24 hours was associated with less persistent organ failure and shorter length of stay, but had no effect on survival [21]. Our study extends these findings in several important ways. First, by including all severity grades (Grade I-III), we provide severity-stratified timing recommendations rather than examining only severe or critically ill cases. Second, our use of discharge disposition as a patient-centered functional recovery outcome addresses a dimension not captured by mortality or length of stay alone. Third, the analysis of multiple time cutoffs (12, 24, 36, and 48 hours) offers more nuanced guidance for clinical decision-making across severity grades.

For Grade I cholangitis, targeting drainage within 24 hours may optimize functional outcomes. The lack of additional benefit from earlier drainage (within 12 hours) indicates that emergency procedures might be unnecessary for stable patients during off-hours. For Grade II, ensuring drainage within 48 hours appears important for survival, supporting guideline emphasis on prompt intervention [3]. On the other hand, early drainage did not improve functional recovery, likely due to age-related frailty and comorbidities inherent in the condition that limit recovery potential [22]. For Grade III, immediate drainage remains the standard as previous studies have shown [8,23], though our limited data preclude specific timing recommendations. The lack of mortality benefit likely reflects the profound physiological compromise at presentation [24]. Across all grades, delays beyond 48 hours were associated with lower functional recovery and survival outcomes. The gradient decline in discharge to pre-admission residence rates with delayed drainage suggests that timely source control prevents the physiological deterioration that can impair recovery even in mild cases [3,8]. The finding supports the principle that earlier drainage generally yields better outcomes [5]. 

This study has several important limitations. First, the observational design precludes causal inference, and unmeasured confounding is likely to affect our estimates. Sicker patients within each grade may have received earlier drainage, potentially biasing results toward the null. Second, we could not perform multivariable adjustment due to limited outcome events, thereby preventing control for important confounders such as age, comorbidities, and etiology. Third, discharge disposition, as a proxy for functional outcome, has limitations. Non-medical factors, including insurance coverage, family support, and facility availability, influence discharge planning. However, this outcome remains clinically relevant and patient-centered. Additionally, the use of validated functional assessment tools such as the Katz Index of Activities of Daily Living [25] or the Barthel Index [26] was not feasible due to the retrospective design and the absence of routine documentation of these measures. Fourth, a single-center design limits generalizability, particularly given local practice patterns and population characteristics. Fifth, we excluded patients receiving percutaneous or surgical drainage, as these patients may represent a distinct severity spectrum. Sixth, some subgroup analyses involve relatively small sample sizes, particularly the Grade III delayed drainage group (n = 10), which limits statistical power and the robustness of those comparisons. Seventh, potential information bias from retrospective data extraction cannot be excluded, although the use of five independent reviewers with consensus resolution mitigates this concern. Additional limitations include potential selection bias from excluding repeat procedures, the absence of long-term functional or quality-of-life outcome assessments, and the inability to assess the impact of specific antimicrobial regimens or supportive care measures.

Future research should include prospective multicenter studies with pre-specified drainage protocols, adequate power for multivariable analysis, incorporation of standardized functional assessment tools (e.g., Katz Index of Activities of Daily Living, Barthel Index), long-term functional outcomes, quality-of-life assessments, and economic evaluations. 

## Conclusions

In this retrospective observational cohort study, associations between biliary drainage timing and outcomes in acute cholangitis varied by severity grade and outcome type. While these findings suggest that severity-stratified drainage protocols considering both survival and functional outcomes may be beneficial, prospective studies with appropriate adjustment for confounders and objective functional measures are much needed to confirm these associations and establish evidence-based timing thresholds.
